# A Cell Proliferation and Inflammatory Signature Is Induced by Lawsonia intracellularis Infection in Swine

**DOI:** 10.1128/mBio.01605-18

**Published:** 2019-01-29

**Authors:** Fernando L. Leite, Juan E. Abrahante, Erika Vasquez, Fabio Vannucci, Connie J. Gebhart, Nathan Winkelman, Adam Mueller, Jerry Torrison, Zachary Rambo, Richard E. Isaacson

**Affiliations:** a Department of Veterinary and Biomedical Sciences, College of Veterinary Medicine, University of Minnesota, Saint Paul, Minnesota, USA; b University of Minnesota Informatics Institute, University of Minnesota, Minneapolis, Minnesota, USA; c Department of Veterinary Population Medicine, College of Veterinary Medicine, University of Minnesota, Saint Paul, Minnesota, USA; d Swine Services Unlimited, Inc., Rice, Minnesota, USA; e Zinpro Corporation, Eden Prairie, Minnesota, USA; VA Palo Alto Health Care System; USDA-ARS-National Animal Disease Center; Washington State University

**Keywords:** *Lawsonia intracellularis*, cell proliferation, host response, intestinal inflammation, transcriptome

## Abstract

Lawsonia intracellularis causes porcine proliferative enteropathy. This is an enteric disease characterized by thickening of the wall of the ileum that leads to decreased growth of animals and diarrhea. In this study, we investigated the host response to L. intracellularis infection by performing transcriptomic and pathway analysis of intestinal tissue samples from groups of infected and noninfected animals at 14, 21, and 28 days postchallenge. At the peak of infection, when animals developed the most severe lesions, infected animals had higher levels of several gene transcripts involved in cellular proliferation and inflammation, including matrix metalloproteinase-7 (*MMP7*), transglutaminase-2 (*TGM2*), and oncostatin M (*OSM*). Histomorphology also revealed general features of intestinal inflammation. This study identified important pathways associated with the host response in developing and resolving lesions due to L. intracellularis infection.

## Abstract (precis)

Lawsonia intracellularis is among the most important enteric pathogens of swine, and it can also infect other mammalian species. Much is still unknown regarding its pathogenesis and the host response, especially at the site of infection. In this study, we uncovered several novel genes and pathways associated with infection. Differentially expressed transcripts, in addition to histological changes in infected tissue, revealed striking similarities between L. intracellularis infection and cellular proliferation mechanisms described in some cancers and inflammatory diseases of the gastrointestinal tract. This research sheds important light into the pathogenesis of L. intracellularis and the host response associated with the lesions caused by infection.

## INTRODUCTION

Lawsonia intracellularis is a Gram-negative, obligate intracellular bacterium that infects enterocytes of swine, primarily in the ileum, and causes a disease called proliferative enteropathy (PE) (1). Infected enterocytes undergo hyperplasia, and macroscopic lesions are marked by thickening of the intestinal mucosa (1). PE is endemic in swine herds worldwide (1, 2), and L. intracellularis has also been shown to infect horses, hamsters, dogs, and nonhuman primates, among other species (3).

In swine, there are two major clinical forms of disease: proliferative hemorrhagic enteropathy (PHE) and porcine intestinal adenomatosis (PIA). PIA is a persistent but self-limiting disease that occurs in young pigs and can lead to diarrhea and reduced growth and is commonly a subclinical disease (1). PHE occurs in older finisher pigs, gilts, and sows, is characterized by hemorrhagic diarrhea, and often leads to death (1). PIA is the most common form of the disease (3) and was the focus of this study.

There is limited knowledge on the pathogenesis of L. intracellularis. This gap in knowledge is partly due to the fastidious nature of the organism, the difficulty growing pure cultures of the organism, and the lack of *in vitro* models that replicate proliferative lesions (3). Similarly, much is still unknown about the mucosal immune response to L. intracellularis. Elevated levels of tumor necrosis factor alpha (TNF-α) and transforming growth factor β (TGF-β) have been found in the infected intestinal mucosa (4, 5), along with an antigen-specific IgA response (6). Infiltration of inflammatory cells, particularly neutrophils, is not a primary feature of infection (1), although accumulation of macrophages has been found to coincide with increased lesion severity and antigen load in this disease (7, 8). Smith et al. (8) analyzed gene expression by microarray analysis at different time points after experimental infection. Interesting patterns were found regarding how L. intracellularis affects mucosal integrity, which confirmed the association of macrophage transcripts with lesions. However, limited information was generated regarding possible mechanisms and pathways responsible for the hallmark lesion of hyperplasia that occurs with disease.

The objective of this study was to investigate the porcine host response to L. intracellularis at the site of infection to gain a better understanding of the pathogenesis and immune response by correlating the presence and severity of lesions with the differential expression of host genes at several time points using RNA-seq and pathway analysis. Our results demonstrated that several gene transcripts associated with cell proliferation and inflammation are differentially expressed in infected animals, a pattern which is exacerbated with increased lesion severity, indicating their likely role in this disease.

## RESULTS

### Gross and microscopic pathology.

Animals developed different levels of lesions and were grouped in those that developed “low” or “high” lesions and level of infection. “Low” lesions were defined as those that had immunohistochemistry (IHC) and hematoxylin and eosin (H&E) microscopic lesion scores of one or zero with or without the presence of gross lesions in the group that received the challenge. “High” lesion animals were defined as those that had an IHC score of 2.5 and above, H&E score of 2 and above in addition to the presence of gross lesions. At 14 days postinfection (dpi), all infected animals had IHC and H&E stain scores of 1, indicating low-level infection with minor lesions, and no animals had gross lesions (Table 1). At 21 dpi, three of six animals (animals 297, 1381, and 97) had H&E stain scores of 3, indicating diffuse microscopic lesions, and the same three animals also had IHC scores of 3 and above, indicating high levels of L. intracellularis bacteria present in the tissue. The other infected animals necropsied at this time point (144, 173, and 192) had IHC and H&E stain scores of 1, indicating minor (low) lesions, and one of these animals had mild gross lesions. All three animals with H&E stain and IHC scores above 2 at 21 dpi had gross lesions, and one of these animals had severe gross lesions (Table 1). At 28 dpi, three animals (94, 197, and 194) had low lesions with either a negative score or a score of one for IHC and H&E stain. The other three animals necropsied at this time point (1386, 1385, and 189) had high lesions, as measured by IHC and H&E stain scores above 2, and moderate or mild gross lesions (Table 1). None of the noninfected animals had microscopic lesions observed by H&E stain or IHC. One animal in the noninfected group necropsied at 28 dpi had a gross lesion score of 1 with mild thickening in Peyer’s patches and hyperemic folds (data not shown).

**TABLE 1:** Measures of infection by *L. intracellularis* at different times postinfection^*a*^

Day postinfection	Animal	Score for indicated assay^*b*^			*C_T_* value	Serum antibody titer^*c*^
		IHC	H&E stain	GL		
14	139	1	1	0	20.02	Neg
	99	1	1	0	26.33	1:30
	148	1	1	0	29.44	Neg
	77	1	1	0	34.97	1:30
	188	1	1	0	26.89	1:30
	87	1	1	0	27.54	1:30

Avg		1.00	1.00	0.00	27.53	1:20

21	144	1	1	0	27.11	1:480
	173	1	1	0	25.51	1:240
	192	1	1	1	26.3	Neg
	297	3*	3*	1	27.08	1:240
	1381	4*	3*	3	17.33	1:240
	97	3*	3*	1	25.04	1:240

Avg		2.17	2.00	1.00	24.73	1:240

28	94	0	1	1	Neg	1:240
	197	0	0	1	Neg	Neg
	194	1	1	1	32.52	1:1,920
	1386	2.5*	3*	2	26.61	1:1,920
	1385	2.5*	2*	1	28.62	1:480
	189	4*	3*	2	23.61	1:960

Avg		1.67	1.67	1.33	27.84	1:920

a The measures of infection include immunohistochemistry, microscopic (H&E stain) and gross lesion scores, *C_T_* value from PCR, and serum antibody titer. Neg, negative result; *, high lesion.

b IHC, immunohistochemistry of *L. intracellularis* antigen in tissue; H&E, hematoxylin and eosin stain of microscopic lesions; GL, gross lesion score.

c Serum antibody titer was measured using the immunoperoxidase monolayer assay (IPMA).

### Shedding and serologic responses.

The results of fecal PCR and the immunoperoxidase monolayer assay (IMPA) serologic assay are shown in Table 1. Animals shed more L. intracellularis bacteria at 21 dpi, when cycle threshold (*C_T_*) values were the lowest among all three time points (*C_T_* value of 24.73 at 21 dpi versus 27.53 at 14 dpi and 27.84 at 28 dpi). At 28 dpi, two animals in the infected group did not shed detectable levels of L. intracellularis in feces. In the noninfected group, two animals shed L. intracellularis (both with a *C_T_* value of 33). Since these animals did not have microscopic lesions measured using H&E stain or IHC and did not have positive antibody responses, they were still included in the study. Antibodies against L. intracellularis were not detected in serum samples of any of the animals in the noninfected group. Five of the six animals in the infected group had detectable antibody titers at 21 and 28 dpi. Antibody titers ranged from an average of 1:20 at 14 dpi to 1:920 at 28 dpi (Table 1).

### Histomorphology.

**FIG 1:**
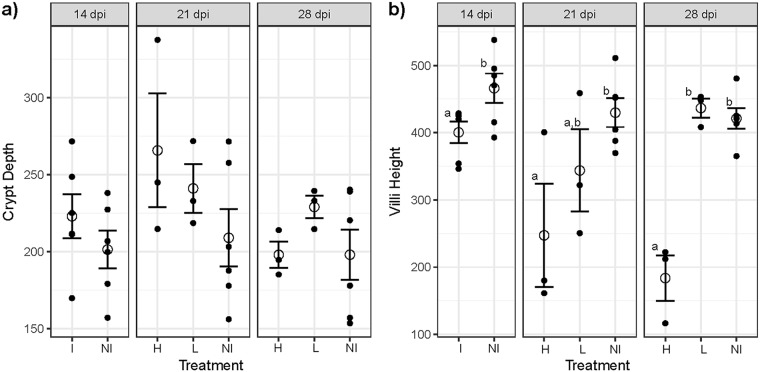
Histomorphology of ileum tissue. (a) Crypt depth. (b) Villus height. Reported are the mean values and standard errors for each treatment by day. Different letters indicate statistical significance (*P < *0.05). Error bars represent standard errors of the means, and open circles indicate the mean values. dpi, days postinfection; I, infected; NI, noninfected; H, high lesion; L, low lesion.

Analysis of crypt and villus morphology revealed that infection affected the intestinal structure, and animals with high lesions responded differently than those with low lesions. Although not statistically significant, infected animals on average had larger crypts at 14 dpi, which peaked at 21 dpi in high-lesion animals compared to those in low-lesion and noninfected animals. At 28 dpi, high-lesion and noninfected animals had similar crypt depths (Fig. 1a). Villus height was also affected by infection. At 14 dpi, villus height was lower in infected animals (*P < *0.05) (Fig. 1b). At 21 dpi, animals in the high-lesion group had the shortest villus height, which was significantly shorter than that in the noninfected group (*P < *0.05) (Fig. 1b). While crypt depth was similar at 28 dpi, villus height was not, and high-lesion animals had significantly lower villus height than other infected and noninfected animals (*P < *0.05) (Fig. 1b).

### DEGs between infected and noninfected animals.

To determine whether tissue lesions corresponded to differences in the abundance profile of gene transcripts expressed in the ileal samples, RNA-seq was employed to compare mRNA profiles between challenged and nonchallenged pigs and between pigs with high and low lesions due to L. intracellularis infection. When we compared samples from challenged and nonchallenged pigs, no altered transcript abundance profiles were observed at 14 days postchallenge. However, at 21 dpi, there were 22 differentially expressed genes (DEGs), while at 28 dpi, only a single DEG was identified.

Among the 22 DEGs at 21 dpi, two were involved in nucleotide metabolism, xanthine dehydrogenase (*XDH*) and cytidine deaminase (*CDA*) (Table 2). The gene encoding gamma-aminobutyric acid receptor subunit pi (*GABRP*) was also among the genes with the greatest transcript abundance. Two matrix metalloproteinase (MMPs) gene transcripts were more abundant; these were *MMP7* and *MMP13*.

**TABLE 2:** Differentially abundant gene transcripts identified at 21 days postinfection comparing infected to noninfected pigs

Ensembl ID	Gene symbol	Fold change	Corrected *P* value
ENSSSCT00000009326.2	*XDH*	816.98	0.009
ENSSSCT00000016344.2	*MMP7*	409.6	0.014
ENSSSCT00000026800.1	*GABRP*	277.33	0.024
ENSSSCT00000014604.2		198.35	3.66E−04
ENSSSCT00000005396.2	*SERPINB2*	128.98	0.009
ENSSSCT00000014601.3	*SAA1*	92.3	0.024
ENSSSCT00000004241.1	*TACSTD2*	58.54	0.016
ENSSSCT00000010382.2		28.02	0.009
ENSSSCT00000029653.1		25.13	0.034
ENSSSCT00000009811.2	*AMCF-II*	23.57	0.009
ENSSSCT00000000312.2		23.06	0.009
ENSSSCT00000010954.2	*OSM*	19.87	0.015
ENSSSCT00000011519.2	*SFRP5*	18.66	0.026
ENSSSCT00000007675.2	*IDO1*	17.62	0.016
ENSSSCT00000000001.2		15.66	0.024
ENSSSCT00000016351.2	*MMP13*	15.04	0.021
ENSSSCT00000005162.1	*DUOXA2*	13.56	0.009
ENSSSCT00000031001.2	*TGM2*	11.72	0.035
ENSSSCT00000005163.2	*DUOXA1*	10.24	0.014
ENSSSCT00000026673.2	*CDA*	7.3	0.013
ENSSSCT00000015915.1		4.21	0.009
ENSSSCT00000010573.2	*ADAMDEC1*	−4.61	0.014

Several of the other DEGs are known to have roles in inflammatory responses, including genes encoding serum amyloid 1 (*SAA1*), whose inducible expression is a hallmark of the acute-phase response (9), oncostatin M (*OSM*), a cytokine involved in diseases with chronic inflammation (10), secreted frizzled-related protein 5 (*SFRP5*), an anti-inflammatory adipokine (11), and transglutaminase-2 (*TGM2*), a functionally complex protein that is induced by inflammatory mediators (12).

Genes described as being expressed by antigen-presenting cells, including macrophages and dendritic cells, were also differentially expressed. These included *SERPINB2*, *IDO1*, *ADAMDEC1*, and *AMCF-II* (encoding alveolar macrophage-derived chemotactic factor II). Other gene transcripts with greater abundance at 21 days postinfection included the tumor-associated calcium signal transducer gene (*TACSTD2*) and the gene transcripts that code for dual oxidase maturation factor 1 and 2 (*DUOXA2* and *DUOXA1*).

The single gene transcript differentially expressed at 28 dpi encoded UNC-5 netrin receptor B (*UNC5B*). Its expression level was 3.77-fold higher in infected animals (*P* < 0.05) than in noninfected animals.

### DEGs between animals with high and low lesions.

**FIG 2:**
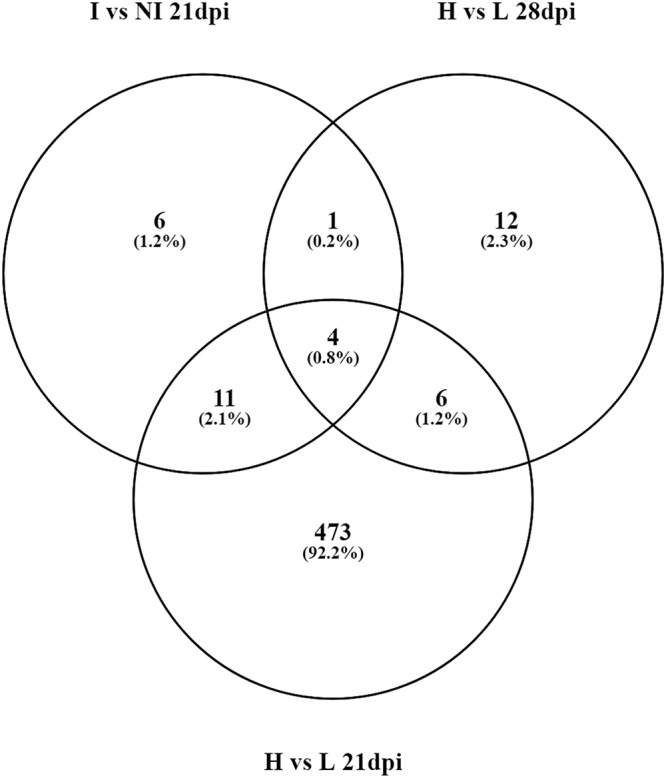
Venn diagram of differentially abundant gene transcripts compared between infected and noninfected animals at 21 dpi (I versus NI 21 dpi), high- and low-lesion animals at 21 dpi (H versus L 21 dpi), and high- and low-lesion animals at 28 dpi (H versus L 28 dpi).

Because pigs within the challenged group at 21 dpi were classified by severity of infection (low versus high lesions), we wanted to know if host gene transcript expression was different between these two groups. Therefore, animals with high lesions were compared to those with low or minimal lesions. With this comparison, there were many more differentially expressed genes (*n* = 494) observed than when comparing all infected animals together to noninfected animals (*n* = 22). At 28 dpi, the comparison between high- and low-lesion animals was different than that at 21 dpi, because two of the three low-lesion animals had either a negative H&E stain score or a negative IHC score and did not shed L. intracellularis at this time point, suggesting they had cleared infection or had a minimal level of infection. At 28 dpi, there were 23 DEGs when comparing animals with high lesions to those with low or no lesions. Figure 2 shows a Venn diagram comparing the numbers of DEGs between groups of high- and low-lesion animals and infected and noninfected animals at different time points.

We focused on the time point of 21 dpi because this is the time point when the peak of infection occurred, as measured by fecal shedding, the number of animals with lesions, and lesion severity. The 10 most increased and the 10 most decreased gene transcripts identified when comparing animals with high lesions to those with low lesions at 21 dpi are shown in Table 3. Similar to the case for DEGs identified comparing challenged to nonchallenged animals at 21 dpi, *MMP7*, *GABRP*, and *SERPINB2* were also among the most differentially expressed genes. Other increased gene transcripts included those encoding adhesion G protein-coupled receptor A2 (*ADGRA2*), bradykinin receptor B1 (*BDKRB1*), TNF-α-induced protein 6 (*TNFAIP6*), and integrin subunit alpha 8 (*ITGA8*). Among the most decreased gene transcripts in high- compared to low-lesion animals at 21 dpi were those of two genes that encode the apolipoproteins C-III (*APOC3*) and A-IV (*APOA4*) and the gene that encodes fibroblast growth factor 19 (*FGF19*). These code for proteins which are expressed in the small intestine and play important roles in lipid metabolism, among other functions (13–15).

**TABLE 3:** The 10 most increased and the 10 most decreased transcripts at 21 dpi comparing animals with high and low lesions^*a*^

Ensembl ID	Gene symbol	Fold change	Corrected *P* value
ENSSSCT00000016344.2	*MMP7*	1129.1	1.04E−05
ENSSSCT00000027635.1	*SERTAD4*	833.28	5.29E−05
ENSSSCT00000026800.1	*GABRP*	762.53	1.08E−04
ENSSSCT00000005396.2	*SERPINB2*	402.41	6.44E−14
ENSSSCT00000017853.2	*TNFAIP6*	342.92	4.13E−03
ENSSSCT00000002779.1	*BDKRB1*	330.73	1.12E−02
ENSSSCT00000000258.2		311.53	4.92E−03
ENSSSCT00000012090.2	*ITGA8*	244.63	9.47E−05
ENSSSCT00000005307.1	*GREM1*	230.45	2.62E−02
ENSSSCT00000017233.2	*ADGRA2*	217.36	1.00E−02
ENSSSCT00000028967.1		−16.92	0.017351
ENSSSCT00000010414.2		−17.93	0.000002
ENSSSCT00000012033.2	*AQP7*	−20.45	0.000621
ENSSSCT00000010411.2		−20.46	0.011745
ENSSSCT00000003670.2	*HELZ2*	−22.44	0.045878
ENSSSCT00000009346.2	*PLB1*	−23.42	0.006509
ENSSSCT00000016435.2	*APOC3*	−42.21	0.000568
ENSSSCT00000016434.2	*APOA4*	−62.85	4.63E−05
ENSSSCT00000014068.2	*FGF19*	−63.33	0.037558
ENSSSCT00000022909.1		−239.38	0.049848

a There were 417 increased transcripts and 77 decreased transcripts.

Among the 23 transcripts that were differentially abundant between high- and low-lesion animals at 28 dpi (Table 4), 4 were in common between infected and noninfected animals, as well as between high- and low-lesion animals at 21 dpi (Fig. 2). These were for the genes *XDH*, *MMP7*, and *TGM2* and a porcine gene with no known human or mouse homologue (ENSSSCT00000014604.2). *IDO1*, which encodes indoleamine 2,3-dioxygenase, was increased in infected animals at 21 dpi, as well as in high-lesion animals at 28 dpi. The transcripts for the gene *MGP*, as well as those of genes *TMEPAI* and *HTRA3*, which have been found to play roles in apoptosis and influencing cell proliferation, were increased in high-lesion animals at both 21 and 28 dpi (16, 17). Around half of the 23 DEGs of high-lesion animals at 28 dpi were only found at that time point. Several of these genes encode proteins involved in antibody biosynthesis, including *IGLC1*, which encodes the constant region of immunoglobulins, and *IGLV-10* and *IGLV-12*, which encode the variable regions of the lambda light chain of immunoglobulins (18). The only gene with a decreased level of transcripts in animals with high lesions at 28 dpi was *CA1*, which codes for carbonic anhydrase 1 (19).

**TABLE 4:** Differentially abundant gene transcripts between high- and low-lesion animals at 28 days postinfection

Ensembl ID	Gene symbol	Fold change	Corrected *P* value
ENSSSCT00000009326.2	*XDH*	522.4	0.013
ENSSSCT00000016344.2	*MMP7*	285.18	0.046
ENSSSCT00000014806.2	*ADGRE1*	276.6	0.006
ENSSSCT00000014604.2		191.45	3.92E−05
ENSSSCT00000004480.2	*FNDC1*	96.61	0.046
ENSSSCT00000013521.2	*VSIG4*	49.46	0.026
ENSSSCT00000016670.2	*STEAP4*	44.33	0.02
ENSSSCT00000027321.1	*FXYD1*	35.54	0.031
ENSSSCT00000008138.2	*PLTP*	30.24	0.031
ENSSSCT00000034787.1	*IGLV-12*	26.57	0.008
ENSSSCT00000000652.2	*MGP*	22.07	0.009
ENSSSCT00000009543.2	*HTRA3*	20.22	0.046
ENSSSCT00000005438.2	*CILP*	20	0.053
ENSSSCT00000031001.2	*TGM2*	19.23	0.026
ENSSSCT00000007675.2	*IDO1*	18.43	0.01
ENSSSCT00000033135.1	*IGLV-10*	14.65	0.01
ENSSSCT00000010999.3	*IGLC*	13.24	3.92E−05
ENSSSCT00000032543.1	*FCN2*	11.78	0.006
ENSSSCT00000010515.2	*ROR2*	11.7	0.046
ENSSSCT00000003426.2	*APOE*	8.51	0.01
ENSSSCT00000008224.1	*TMEPAI*	5.2	0.046
ENSSSCT00000006735.2	*CA1*	−44.61	0.046
ENSSSCT00000022705.1		−166.98	0.004

### Pathway analysis. (i) Biological functions associated with increased lesion severity.

To explore potential biological functions associated with the hyperplasia that is characteristic of L. intracellularis infection, biological functions associated with “proliferation” were investigated. This pathway analysis revealed 16 biological functions significantly associated (*P < *0.05) with the DEGs, and the most significant was “cell proliferation of tumor cell lines” (*P = *1.4E−15) (Table 5). Ingenuity Pathway Analysis (IPA) attributes an activation z-score that assesses the randomness of directionality within a gene set to infer the activation state of a metric (i.e., biological function, upstream regulator, or pathway) as either activated or inhibited. A negative z-score indicates inhibition and a positive z-score indicates activation, with scores equal to or greater than 2 or equal to or less than −2 being statistically significant (*P < *0.05) (20). Of the 16 biological functions associated with proliferation, 6 had significant z-scores of above 2, including “cell proliferation of tumor cell lines” (Table 5).

**TABLE 5:** List of diseases and functions associated with “proliferation” among the diseases and functions identified between animals with high and low lesions using Ingenuity Pathway Analysis

Function annotation	*P* value	z-score	No. of molecules
Proliferation of connective tissue cells	4.75E−14	3.983	50
Cell proliferation of fibroblasts	4.32E−09	3.873	29
Proliferation of synovial cells	5.92E−08	2.922	9
Cell proliferation of tumor cell lines	1.4E−15	2.671	98
Proliferation of stem cells	0.000014	2.52	16
Cell proliferation of breast cancer cell lines	0.000000235	2.075	32
Proliferation of pericytes	0.0000125	1.71	8
Endothelial cell development	0.000000131	1.461	27
Proliferation of liver cells	0.0000153	1.25	15
Proliferation of tumor cells	0.0000106	1.22	28
Proliferation of endothelial cells	9.05E−08	1.142	25
Proliferation of epithelial cells	7.77E−09	1.007	36
Cell proliferation of ovarian cancer cell lines	0.00000357	0.891	13
Colony formation of cells	4.52E−08	0.429	34
Colony formation	1.85E−08	0.401	37
Colony formation of tumor cell lines	0.00000122	−0.537	22

### (ii) Canonical pathways associated with increased lesion severity.

Next, we used IPA to identify canonical pathways associated with the DEGs between high- and low-lesion animals at 21 dpi. Of these pathways, we searched for pathways with a significant activation status. This analysis revealed 10 significantly activated canonical pathways associated with the data set (z-score of ≥2) (Table 6).

**TABLE 6:** Activated canonical pathways in animals with high lesions compared to low-lesion animals at 21 days postinfection

Canonical pathway	−log(*P* value)	z-score	No. of molecules
Osteoarthritis pathway	8.31	2	19
Oncostatin M signaling	3.62	2.236	5
Estrogen-mediated S-phase entry	3.2	2	4
Aryl hydrocarbon receptor signaling	2.49	2.646	8
Leukocyte extravasation signaling	2.43	3.162	10
Dendritic cell maturation	1.79	2.828	8
Wnt/Ca^+^ pathway	1.72	2	4
Acute-phase response signaling	1.59	2.236	7
Chemokine signaling	1.43	2	4
Melatonin signaling	1.38	2	4

### (iii) Upstream regulators of differentially expressed genes.

Potential regulator molecules responsible for the differential gene expression of the different pathways observed at 21 dpi were identified using IPA. Upstream regulators are molecules that are known to influence the observed DEGs and are potentially responsible for modulating their differential expression (20). Cyclin-dependent kinase inhibitor 1A (CDKN1A, *P* = 2.06E−36), tumor protein p53 (TP53, *P* = 9.48E−29), and Erb-b2 receptor tyrosine kinase 2 (ERBB2, *P = *2.57E−26) were among the upstream regulators most significantly associated with the data set. Other significant upstream regulators included the cytokines transforming growth factor beta 1 (TGFB1, *P = *3.51E−28), tumor necrosis factor (TNF, *P = *2.23E−23), interleukin-1β (IL-1β), and IL-6 (Table 7).

**TABLE 7:** The 30 most significant upstream regulators associated with the differentially expressed genes between animals with high and low level of infection

Upstream regulator	Fold change	Molecule type	z-score	*P* value
CDKN1A		Kinase	−2.696	2.06E−36
TP53		Transcription regulator	−1.797	9.48E−29
TGFB1		Growth factor	4.163	3.51E−28
ERBB2		Kinase	4.368	2.57E−26
TNF		Cytokine	2.31	2.23E−23
Calcitriol		Chemical—drug	−2.059	1.12E−20
HGF		Growth factor	3.546	1.94E−20
RABL6		Other	4.472	4.4E−20
Lipopolysaccharide		Chemical—drug	4.252	1.22E−19
CCND1		Transcription regulator	2.339	2.05E−19
Beta-estradiol		Chemical—endogenous mammalian	3.237	4.04E−19
Tretinoin		Chemical—endogenous mammalian	2.096	6.88E−19
Vegf		Group	4.611	1.04E−18
PDGF BB		Complex	3.059	1.07E−18
IL6		Cytokine	3.836	1.88E−18
dextran sulfate		Chemical—drug	1.627	3.7E−18
SMAD7		Transcription regulator	−1.759	5.52E−18
Medroxyprogesterone acetate		Chemical—drug	−2.449	5.52E−18
E2F4		Transcription regulator	−0.927	3.52E−17
Alpha catenin		Group	−4.145	9.43E−17
PTGER2		G-protein coupled receptor	3.702	1.32E−16
IL-1β	29.3	Cytokine	3.216	1.53E−16
TGF-β		Group	1.365	3.53E−16
Cycloheximide		Chemical—reagent	0.371	5.29E−16
Dexamethasone		Chemical—drug	0.526	1.41E−15
EP400		Other	3.148	2.43E−15
CDKN2A		Transcription regulator	−2.69	5.15E−15
Butyric acid		Chemical—endogenous mammalian	−0.573	8.37E−15
Trichostatin A		Chemical—drug	1.112	1.77E−14
MYC	4.2	Transcription regulator	2.574	2.78E−14

### MMP-7 immunohistochemistry

**FIG 3:**
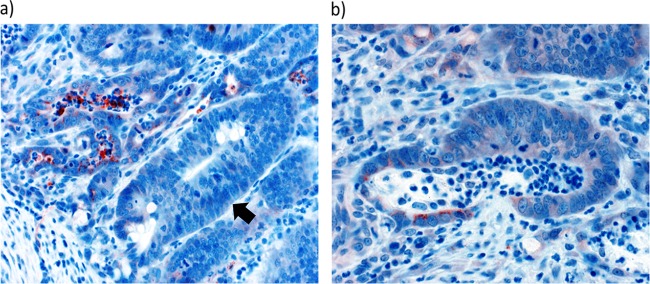
Matrix metalloproteinase-7 (MMP-7) staining. (a) Luminal staining of MMP-7 (red) in crypts adjacent to a hyperplastic crypt (arrow). (b) Apical staining of MMP-7 (red) enterocytes in crypt.

To determine if changes observed at the level of transcription of *MMP7* were translated into protein in infected tissue, we chose to perform immunohistochemistry staining for matrix metalloproteinase-7 (MMP-7). We observed that the distribution of staining of this molecule differed between infected and noninfected animals. Staining of MMP-7 was not associated with hyperplastic crypts. MMP-7-positive cells were observed in normal crypts adjacent to those undergoing hyperplasia. Staining was observed both in the apical side of enterocytes and associated with the lumen of crypts (Fig. 3). This pattern of staining was not observed in noninfected animals (data not shown).

## DISCUSSION

In this study, we reproduced PPE with the induction of characteristic microscopic and macroscopic lesions. We also assessed clinical signs, and as in most cases of PIA in the field, animals had subclinical disease (data not shown). Infection was also confirmed by quantifying L. intracellularis bacteria shed in feces and the presence of antibodies in serum. We observed a time effect of disease that has previously been described in which hyperplasic lesions begin at around 11 dpi, peak at around 21 dpi, and begin to resolve at 28 dpi (21). Changes in crypt depth, fecal shedding, and villus height were observed and were more severe at 21 dpi in infected animals. This is in accordance with previous studies and suggests that the peak of infection occurred at this time point. At 28 dpi, two of the six animals had either a negative IHC score or a negative H&E stain score and did not shed L. intracellularis, indicating a likely resolution of infection, as is known to occur in this disease (1). At 28 dpi, animals also showed high antibody titers against L. intracellularis, indicative of a strong prior immune response to infection.

In this study, at 14 dpi, the lack of differentially expressed genes coincided with low lesion severity and antigen load. This was also observed by Smith et al. (8), who analyzed L. intracellularis-infected intestine tissue samples at different time points by microarray analysis and found that the number of DEGs coincided with the quantity of L. intracellularis bacteria present in tissue. This indicates that there is not a robust host response during early stages of the infection, and it coincides with lower lesions. At 21 dpi, when animals developed severe lesions, there were 22 DEGs between infected and noninfected animals. When comparing animals with high and low levels of infection and high and low lesions at this time point, there were 494 DEGs, again suggesting that the development of severe lesions is associated with a greater host response. Several of the genes found to be differentially expressed in this study were not observed by Smith et al. (8), although “cancer” was the major disease category found with pathway analysis in both studies (data not shown; *P = *5.86E−23 to 1.9E−05). Differences in gene expression between both studies may be due to differences in lesion severity caused by infection and/ordifferences between microarray and RNA-seq methods of gene expression analysis.

The transcript most highly and differentially abundant in infected animals at 21 dpi was *XDH* (816.98-fold higher in infected animals; *P = *0.009) (Table 2), which encodes xanthine dehydrogenase. This gene was also differentially expressed between high- and low-lesion animals at both 21 and 28 dpi. Xanthine dehydrogenase is a key regulator of inflammatory cascades, whose products include reactive oxygen and nitrogen species (22, 23). This enzyme is highly expressed in the small intestine and has antimicrobial properties (23). Recently, it was found to be highly expressed in Mycobacterium avium subspecies *paratuberculosis* infection, where it was suggested to be indicative of macrophage activation and killing of the pathogen. A similar response is possible in infections with L. intracellularis (24).

*MMP7*, which encodes matrix metalloproteinase-7, or matrilysin, was also a transcript that was found to be upregulated in infected animals and in animals with high lesion scores at 21 and 28 dpi. This protein can be induced by some Gram-negative bacteria, and it has an important role in activating α-defensin antimicrobial peptides (25, 26). Pathogenic strains of Helicobacter pylori, which have a greater capacity to cause gastric cancer, induce *MMP7* expression in a *cag* pathogenicity island-dependent manner (27, 28). Overexpression of *MMP7* is commonly found in colorectal cancers, and this protein can induce increased cellular proliferation (29–32). Interestingly, positive staining of this protein in ileal tissues from animals with high lesions was found in crypts adjacent to those undergoing hyperplasia (Fig. 3).

Among the diseases and functions associated with the DEG data set involving proliferation, the biological function of “cell proliferation of tumor cell lines” was the most significantly associated with the data set and had a significantly activated status (*P = *1.4E−15, z-score of 2.67). This function had the involvement of 98 of the 494 DEGs among animals with high lesion scores at 21 dpi, which also correlated with the time when peak of crypt depth was noted using histomorphometry (Fig. 1a). The transcripts of genes *MMP7*, *OSM*, *SERPINB2*, *GABRP*, *TACSTD2*, and *TGM2*, which were differentially abundant between infected and noninfected animals at 21 dpi, were also among these 98. This demonstrates that the molecular signature induced by L. intracellularis is strongly associated with cellular proliferation, several molecules of which have also been found to be upregulated in different tumor cell lines. This makes sense, since hyperplasia of enterocytes is observed in L. intracellularis infection. The two upstream regulators most significantly associated with the data set, cyclin-dependent kinase inhibitor 1A (CDKN1A, *P* = 2.06E−36) and tumor protein p53 (TP53, *P* = 9.48E−29), are also associated with cell proliferation. CDKN1A inhibits the cyclin kinases that are necessary for cell cycle progression during the G_1_ and S phases and has an antiproliferative effector function. This gene is induced by p53 tumor suppressor protein, which is activated upon encounter with cellular stressors to induce the expression of genes that result in inhibition of proliferation (33, 34). Both of these regulators had negative z-scores, indicating that they were downregulated and, thus, likely not maintaining their function of inhibiting cell proliferation (Table 7).

There is a large body of evidence to suggest that tumor-associated calcium signal transducer 2 (also named Trop2; encoded by *TACSTD2*) promotes cellular proliferation, and this gene is highly expressed in a wide variety of epithelial tumors (35, 36). Interestingly, it has also been found that knockdown of this gene in mice can promote carcinogenesis in a squamous cell cancer model, which also suggests a protective role of this protein (37). Similarly, transglutaminase-2 (*TGM2*) can also promote cell proliferation, although this protein has many other functions (38). *TGM2* expression is induced by inflammatory mediators like IL-1β, TNF-α, and TGF-β, and it has been described to be involved in inflammatory conditions of the intestine (12, 39). TNF, IL-1β, and TGF-β were found to be upstream regulators (Table 7) and are likely to contribute to the host response to L. intracellularis. Interestingly, a common histomorphological feature of inflammatory conditions of the intestine is hyperplasia of enterocytes, which is marked by an increase in epithelial cell numbers that are visible as elongated crypts (40). Although not statistically significant, we found that animals with high lesions had greater average crypt depth, demonstrating that crypts are enlarged with more severe infection (Fig. 1a); elongated crypts are a common feature of PPE (1). Two other histomorphological features of intestinal inflammation are villus blunting and loss of goblet cells (40, 41). We also observed a decrease in villus height with infection (Fig. 1b), and the decrease or absence of goblet cells is known to occur in L. intracellularis infection (1). This suggests that the host response to infection may contribute to the altered state of enterocytes, in addition to any potential mechanism L. intracellularis may itself possess.

Also associated with inflammation is the gene *OSM*, which encodes oncostatin M, a member of the IL-6 cytokine family, expressed by activated T cells, monocytes, and dendritic cells (10). Increased expression of this cytokine has been found in inflammatory bowel disease, where it correlates with histopathological disease severity (42, 43). OSM is also capable of inducing enterocyte proliferation (42). In our data set, not only was the *OSM* gene transcript increased with infection, but the oncostatin M signaling canonical pathway was also significantly associated and activated in animals with high lesion scores at 21 dpi (Table 6).

Several of the other canonical pathways and differentially expressed genes were also involved in inflammation and immune response. These include the pathways of leukocyte extravasation signaling, dendritic cell maturation, and acute-phase response signaling. Increased levels of acute-phase proteins have previously been found in pigs infected with L. intracellularis (44). This gives some insight into the mucosal immune response to infection, which is known to be protective, and suggests that antigen presentation with dendritic cells and the presence of leukocytes are important parts of this process. Aryl hydrocarbon receptor (AhR) signaling is becoming increasingly recognized as an important pathway that influences the immune responses to pathogens (45). This pathway was significantly activated in animals with high lesions at 21 dpi (Table 6). The precise function of this signaling pathway varies with different pathogens and affected organs (45). In infections of mice with Citrobacter rodentium, a bacterial pathogen that also infects the gut and induces hyperplasia of enterocytes (46), knockdown of the *AhR* gene led to significant weight loss and death, while control animals did not succumb to infection (47). Perhaps activation of AhR signaling at the peak of infection is protective against L. intracellularis infection and contributes to the resolution of lesions with time.

Investigating the gene transcripts that were differentially abundant exclusively at 28 dpi gives some insight into the host response associated with the start of resolving lesions. The only transcript that was differentially abundant between infected and noninfected animals at this time point was of the gene *UNC5B* (UNC-5 netrin receptor B), which had a 3.77-fold-higher abundance in infected animals (*P < *0.05). This gene encodes a transmembrane receptor that can regulate cell fate as a “dependence receptor” and induces apoptosis in the absence of its ligand (48, 49). Higher expression of *UNC5B* can inhibit cellular proliferation in some cancer cell lines, and this protein is inactive and downregulated in colorectal cancer (48, 50, 51). Additionally, increased expression of *UNC5B* in leukocytes has been shown to attenuate inflammation (52). Thus, higher expression of *UNC5B* may be involved in inhibiting enterocyte hyperplasia and/or resolving inflammation. The only transcript found to be more abundant in low-lesion animals than in high-lesion animals at this time point was of the gene *CA1* (carbonic anhydrase 1 [CA-1]). Carbonic anhydrases can be found along the entire gastrointestinal tract, and in the small intestine, carbonic anhydrase 1 is found in cryptal enterocytes (53). Interestingly, increased expression and staining of CA-1 is found in healthy intestinal mucosa, where it participates in regulation of pH homeostasis and water and ion transport, while decreased CA-1 protein and mRNA levels can be found in benign and malignant colorectal tumors (53–55). Lastly, several of the increased gene transcripts only found in animals with high lesions at 28 dpi were associated with synthesis of immunoglobulins. This gives important insight as to the active immune response that is occurring at this stage of infection.

The research described here leads us to conclude that the host response to infection leads to the induction of several genes and pathways associated with cellular proliferation, with some similarity to what has been described in some cancers and inflammatory diseases of the intestinal tract. However, infections caused by L. intracellularis are very different from cancer, as the proliferative lesions do not develop into tumors and in fact resolve. The mechanisms that lead to the expression of the observed gene transcripts associated with increased cellular proliferation should be investigated further, as well as their specific contribution to disease. The return to a normal state of the intestine without the expression of these genes is likely due to a protective immune response that clears the pathogen. This research sheds important light into the pathogenesis of L. intracellularis, demonstrating several of the genes and pathways associated with its pathology.

## MATERIALS AND METHODS

### Animals and treatments.

The animal protocol used was approved by the Swine Services Unlimited, Inc., Institutional Animal Care and Use Committee, and all experiments were performed in accordance with relevant guidelines and regulations. Genetically, pigs were from a Landrace cross to a Yorkshire female with a Large White sire (Topigs Norsvin). Two groups of 18 animals were used in this study. One group was infected with Lawsonia intracellularis, and the other group was not infected. The inoculum used was prepared following a previously described protocol and consisted of a mucosal homogenate which was negative for other bacterial and viral pathogens, including porcine reproductive and respiratory syndrome virus (56). Animals were challenged with a dose calculated by quantitative PCR (qPCR) to be of 8.0 × 10^7^ L. intracellularis organisms by oral gavage at 7 weeks of age. Groups of 6 pigs per treatment were randomly selected and euthanized at 14, 21, and 28 days postchallenge.

### Gross and microscopic pathology evaluation.

The entire intestinal tract was examined for lesions characteristic of PE. Each macroscopic (gross) lesion was scored as either mild (score of 1), moderate (score of 2) or severe (score of 3). For histopathology and evaluation of microscopic lesions characteristic of PE, a section of ileum proximal to the ileocecal valve was collected from each animal. This section of the intestine was chosen as it has been described as the most consistent site of L. intracellularis infection (1, 7). The sections were then stained using hematoxylin and eosin (H&E) and used for immunohistochemistry (IHC) by staining using murine anti-L. intracellularis-specific polyclonal antibody following previously described protocols (57). The presence of L. intracellularis-specific antigen was measured with a 5-grade IHC scoring scale (grade 0 equal to no L. intracellularis antigen found in tissue, grade 1 equal to 0 to 25%, grade 2 equal to 25 to 50%, grade 3 equal to 50 to 75%, and grade 4 equal to more than 75% enteric crypts containing antigen) (58). The pathologist reading the slides was blinded to which experimental group was being scored. Microscopic lesions in H&E-stained sections were measured using a 4-grade scale representing the distribution of crypt dysplasia (zero for no lesions, 1 for focal lesions, 2 for multifocal lesions, and 3 for diffuse lesion distribution). An animal was considered to have a high level of infection and lesions if the IHC score was 2.5 or above, the H&E stain score was 2 or above, and there was the presence of gross lesions. Animals with a low level of lesions were defined as those that had IHC and H&E stain scores of 1 or zero with or without the presence of gross lesions in the group that received the challenge.

### Histomorphometry.

To assess differences in crypt and villus morphology due to infection, intestinal sections from animals were evaluated by measuring crypt depth and villus height. For this measurement, images were captured from slides using light microscopy with a 10× objective and analyzed with ImageJ (59). Twenty intact and well-oriented villi were randomly selected along with 30 randomly selected crypts for the measurement of their heights and depths, respectively. In instances where 20 villi could not be found, all villi in the image were counted. The average crypt depths and villus heights among high-lesion, low-lesion, and noninfected animals were compared using Tukey’s comparison in an analysis of variance (ANOVA) in R (version 3.3.3 [2017-03-06]) with the level of significance set at 0.05.

### Shedding and serologic responses.

Antibodies against L. intracellularis in serum samples were measured using the immunoperoxidase monolayer assay (IPMA) (60). Fecal shedding of L. intracellularis was measured using qPCR (61).

### Differential gene expression and pathway analysis.

Immediately following necropsy, a section of ileum 2 to 3 cm immediately proximal to the ileocecal junction was opened longitudinally and scraped using the edge of a microscope slide. The sample was immediately placed in RNAlater (Thermo Fisher), and total RNA was extracted using the RNeasy plus universal minikit (Qiagen). RNA quantity and quality were assessed using the RiboGreen RNA assay kit and by capillary electrophoretic sizing using an Agilent TapeStation/Bioanalyze, respectively. Samples that passed quality metrics were used to create a cDNA library for sequencing using an Illumina TruSeq RNA Sample Preparation Kit. Samples from each pig were individually sequenced with the Illumina HiSeq 2500 sequencer using 50 paired-end reads; 20 million reads per sample were obtained. The Illumina sequence files were processed using a pipeline developed by the University of Minnesota Informatics Institute. Briefly, FastQ files were trimmed via trimmomatic and mapping was performed via TopHAT (version 2.0.13) using Bowtie (version 2.2.4.0). The Sus scrofa 3.0 (susScr3) genome was used, and gene annotation was performed using Ensembl from the same genome build. Differentially expressed gene (DEG) analysis was performed using CLC Genomics Workbench (CLCGWB version 9.0.1; Qiagen Bioinformatics) and EdgeR. EdgeR has been described to have superior specificity and sensitivity, as well as good control of false-positive errors, compared to other methods to detect DEGs (62). Ingenuity Pathway Analysis (IPA; Qiagen) was used for pathway analysis.

Comparisons were made between infected and noninfected animals at each time point postinfection, as well as between animals with high- (severe and diffuse) and low-level lesions. At 21 dpi, animals with high lesions (1381 and 97) were compared to the animals with low lesions (144, 173, and 192) (Table 1). RNA could not be extracted from the intestinal sample of animal 297, which had high lesions. At 28 dpi, the high-lesion animals, 1385, 189, and 1386, were compared to animals 94, 197, and 194, which had low lesions.

### Immunohistochemistry of MMP-7.

To measure the presence and distribution of matrix metalloproteinase-7 (MMP-7), which is encoded by the gene *MMP7*, IHC was performed on paraffin-embedded tissue collected at necropsy. IHC of matrix metalloproteinase-7 was performed using a monoclonal antibody against MMP-7 following an adaptation of a previously described protocol (63), using the primary monoclonal antibody against MMP-7 at a dilution of 1:600. The antibody was obtained from Vanderbilt Antibody and Protein Resource.

### Accession number(s).

The entire set of supporting gene expression and genomic data can be found in the Gene Expression Omnibus (GEO) database under accession number GSE122764.
